# Nerve Segmentation with Deep Learning from Label-Free Endoscopic Images Obtained Using Coherent Anti-Stokes Raman Scattering

**DOI:** 10.3390/biom10071012

**Published:** 2020-07-08

**Authors:** Naoki Yamato, Mana Matsuya, Hirohiko Niioka, Jun Miyake, Mamoru Hashimoto

**Affiliations:** 1 Graduate School/Faculty of Information Science and Technology, Hokkaido University, Sapporo 060-0814, Japan; n-yamato@ist.hokudai.ac.jp (N.Y.); m-matsuya@ist.hokudai.ac.jp (M.M.); 2 Institute for Datability Science, Osaka University, Suita 565-0871, Japan; 3 Hitz Research Alliance Laboratory, Graduate School of Engineering, Osaka University, Suita 565-0871, Japan; jun_miyake@bpe.es.osaka-u.ac.jp; 4 Faculty of Information Science and Technology, Hokkaido University, Sapporo 060-0814, Japan

**Keywords:** deep learning, semantic segmentation, nerve imaging, coherent anti-Stokes Raman scattering endoscopy

## Abstract

Semantic segmentation with deep learning to extract nerves from label-free endoscopic images obtained using coherent anti-Stokes Raman scattering (CARS) for nerve-sparing surgery is described. We developed a CARS rigid endoscope in order to identify the exact location of peripheral nerves in surgery. Myelinated nerves are visualized with a CARS lipid signal in a label-free manner. Because the lipid distribution includes other tissues as well as nerves, nerve segmentation is required to achieve nerve-sparing surgery. We propose using U-Net with a VGG16 encoder as a deep learning model and pre-training with fluorescence images, which visualize the lipid distribution similar to CARS images, before fine-tuning with a small dataset of CARS endoscopy images. For nerve segmentation, we used 24 CARS and 1,818 fluorescence nerve images of three rabbit prostates. We achieved label-free nerve segmentation with a mean accuracy of 0.962 and an $F_{1}$ value of 0.860. Pre-training on fluorescence images significantly improved the performance of nerve segmentation in terms of the mean accuracy and $F_{1}$ value (p<0.05). Nerve segmentation of label-free endoscopic images will allow for safer endoscopic surgery, while reducing dysfunction and improving prognosis after surgery.

## 1. Introduction

Recently, endoscopic surgery has attracted significant attention, not only from the medical community, but also the general public, because of its minimal invasiveness [1]. In endoscopic surgery, the surgeon opens several small holes of about 1 cm in diameter in the abdominal cavity, chest cavity, or retroperitoneal cavity, inserts a rigid endoscope and surgical instruments into the body of the patient through the holes, and operates while observing a lesion or affected area under the endoscope. The smaller incisions and faster recovery in endoscopic surgery reduce the burden on patients when compared with conventional open surgery [2,3,4].

Important issues that must be addressed to achieve further advances in endoscopic surgery include visualization and identification of lesions and tissues. Surgeons identify lesions and tissues that are based on slight color differences, shapes, and anatomical knowledge. Even though new staining reagents for visualizing and identifying lesions and tissues have been reported [5,6], no suitable reagents have yet been introduced into clinical settings. Therefore, the development of a label-free visualization method is desired for identifying lesions and tissues, as well as tissues that should be preserved, such as peripheral nerves in nerve-sparing surgery.

The authors have developed a rigid endoscope that utilizes coherent anti-Stokes Raman scattering (CARS) to visualize nerves without staining for endoscopic surgery [7,8]. CARS is a Raman scattering phenomenon that is sensitive to molecular species by utilizing molecular vibrations and achieves label-free molecular imaging [9,10]. CARS is light emission when the frequency difference between two laser beams matches with the frequency of molecular vibration. Since the vibrational frequencies of molecules are sensitive to molecular species, label-free molecular selective imaging is achieved by tuning the frequency difference of two laser beams. In addition, CARS enables faster imaging than the commonly used spontaneous Raman scattering [11]. The CH${}_{2}$ stretching vibrations (2845 cm${}^{-1}$) of saturated or unsaturated aliphatic chains of lipids emit strong CARS. We have demonstrated the visualization of myelinated nerves with the developed endoscope using the CH${}_{2}$ stretching vibration of lipids, because myelin sheaths composed mainly of lipids surround the axon of myelinated nerves.

Automated nerve extraction from CARS endoscopic images by computers will reduce the burden on surgeons in nerve-sparing surgery and contribute to further progress towards safer surgery. Because the CARS signal of the CH${}_{2}$ stretching vibration contains information about all lipids, as well as nerves, it is necessary to distinguish the nerves from other lipid-rich tissues in the image. The training of surgeons is required in order to introduce medical imaging with new modalities. Helping with image interpretation will reduce the burden on surgeons and will contribute to improving the safety of endoscopic surgery.

In the work described in this paper, we demonstrated automated nerve segmentation from CARS images with deep learning. Deep learning is a machine learning technique that builds analysis models automatically through a large dataset and it has undergone rapid development in recent years because it has shown better results compared with humans, especially in the field of image processing [12]. In general, the training of deep learning models requires a large dataset; however, it is difficult to prepare a large number of CARS endoscopy images because CARS endoscopy imaging requires a long time to find nerves. Here, we propose using a model pre-trained with general images and fluorescence images before training with a small dataset of CARS endoscopy images.

## 2. Materials and Methods

### 2.1. Sample Preparation

To observe peripheral nerves, we purchased rabbit urinary organs, including the prostate, from Japan Lamb Co., Ltd. (Fukuyama, Japan). The rabbit prostate tissues were immersed in 4% paraformaldehyde phosphate buffer solution (Fujifilm Wako Pure Chemical, Osaka, Japan) for storage because the formalin fixation has substantially no affection on the CARS signal of the CH${}_{2}$ stretching vibration compared with fresh tissues and cells [13,14]. For imaging, the formalin-fixed rabbit prostate tissues were prepared by washing them with phosphate buffered saline (PBS) for 20 min. The prostates were split laterally for the sample of fluorescence and CARS imaging. After that, peri-prostatic fasciae were cut from the tissues at symmetrical positions around prostates.

### 2.2. Fluorescence Images

The cut fasciae were doubly stained to distinguish between nerves and the other tissues. FluoroMyelin${}^{\mathrm{TM}}$ Green F34651 (Thermo Fisher Scientific, Waltham, MA, USA, Ex/Em: 479/598 nm) stains lipid, allowing for the visualization of the myelin sheath. Hoechst 33342 (Dojindo, Ex/Em: 350/461 nm) was also used for nuclei staining. The staining procedure was as follows. First, 20 μL of FluoroMyelin${}^{\mathrm{TM}}$ Green and 20 μL of Hoechst 33342 were diluted 300-fold in PBS. Second, the fasciae were immersed in the staining solution for 20 min at room temperature. Finally, the fasciae were removed from the solution and ultrasonically washed three times for 10 min each in PBS. Each fascia was sandwiched between cover glasses, and the gap was filled with PBS to prevent drying.

Z-stack fluorescence images of nerves were acquired by a confocal fluorescence microscope (Eclipse Ti, Nikon, Tokyo, Japan). The signals of FluoroMyelin${}^{\mathrm{TM}}$ Green and Hoechst 33342 were detected with the timing shifted-mode. For FluoroMyelin${}^{\mathrm{TM}}$ Green, the wavelength of the excitation light was 488 nm, and the wavelength of the detection light was 605–1000 nm. For Hoechst 33342, the wavelength of the excitation light was 405 nm, and the wavelength of the detection light was 417–477 nm. An objective lens with a magnification of 10-times (Plan Apo, × 10, NA = 0.45, Nikon) was employed. The step width for z-stack imaging was 1 μm. The z-stack images were averaged in the z-direction. The number of pixels was 1024 × 1024, and the imaging area was 1270 × 1270 μm for one image. A total of 153 fluorescence images were acquired.

We divided each image into 16 parts without overlapping, and then removed the images that contained only a small part of nerves and were not distinguished from lipid droplets. A total of 1818 images in which the number of pixels was 256 × 256 and the imaging area was 317 × 317 μm were prepared from one rabbit prostate.

### 2.3. Coherent Anti-Stokes Raman Scattering Rigid Endoscopy Images

Each cut fascia, without staining, was sandwiched between cover glasses, and the gap was filled with PBS to prevent drying.

CARS images of nerves were acquired by the CARS rigid endoscope that we developed. The optical setup of the CARS rigid endoscope has already been described [8]. We obtained a total of 24 CARS images from two rabbit prostates. The CARS images were acquired by two different optical setups. In one setup, we used a lens barrel with a length of 550 mm and an objective lens with a focal length of 7.5 mm. Four CARS images with a wide field of view (635 × 635 μm) were acquired, and each image was divided into four parts. As a result, a total of 16 images with 256 × 256 pixels and an imaging area of 317 × 317 μm were prepared. In the other setup, we used a lens barrel with a length of 270 mm and an objective lens with a focal length of 3.1 mm. Three CARS images with 256 × 256 pixels and an imaging area of 317 × 317 μm were acquired. Five CARS images with 500 × 500 pixels and an imaging area of 262 × 262 μm were also acquired. They were resized to 512 × 512 pixels by symmetric padding, and then halved to 256 × 256 pixels by using the area averaging method. The acquisition time was 330 s and 160 s for the former 19 images and the latter 5 images, respectively.

### 2.4. Architecture and Training of Deep Neural Network

**Figure 1:**
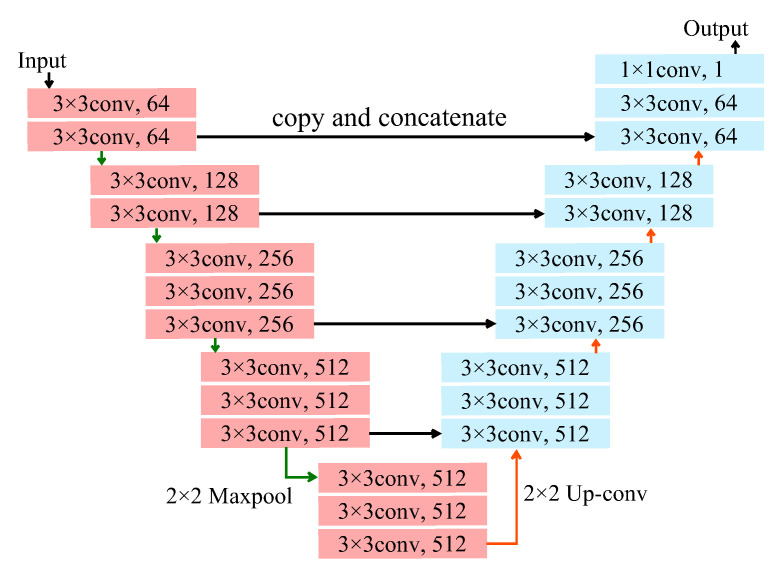
Network architecture of U-Net with VGG16 encoder.

As a deep neural network for semantic segmentation, we employed U-Net with a VGG16 encoder [15]. Figure 1 shows the architecture of U-Net with the VGG16 encoder. U-Net [16] is composed of an encoder part and a decoder part, and VGG16 is used for the encoder part shown in pink in Figure 1. The architecture was constructed by a decoder part with a structure symmetrical to the VGG16 encoder part in a similar manner to U-Net. The final layer outputs the probability of whether each pixel indicates a nerve by using the softmax function, and then the probability images are binarized with a threshold of 0.5.

We used a loss function given by

(1) Loss=1−sensitivity∗specificity,

where

(2) $$sensitivity=\frac{TP}{TP+FN},$$

(3) $$specificity=\frac{TN}{TN+FP}$$

Here, true positive (TP) and true negative (TN) indicate the areas correctly predicted as nerves and background, and false positive (FP) and false negative (FN) indicate the areas wrongly predicted as nerves and background, respectively. Adam optimization [17] was used with $\beta_{1}$ of 0.9 and $\beta_{2}$ of 0.999, and a learning rate of 0.001. He’s initialization method [18] was used to initialize the parameters of untrained layers. Training continued until the number of epochs was 100 or the loss function of validation data was not updated for 20 consecutive epochs. Data augmentation was applied to prevent overfitting. The images were flipped horizontally and vertically and then rotated 90 and 180 degrees. Nerves run in various directions, and a change of the nerve direction will not cause any problems.

The model was implemented with the PyTorch framework [19]. We utilized a desktop computer with a Core i7-8900K processor (Intel) and a GeForce RTX 2080Ti graphic card (NVIDIA).

### 2.5. Evaluation

We conducted multiple test for evaluating the performance of three schemes (Scheme I–III): in Scheme I, U-Net without pre-training was trained on CARS images from scratch, in Scheme II, U-Net with VGG16 encoder was trained on CARS images, in Scheme III, U-Net with VGG16 encoder was fine-tuned on CARS images after pre-training on fluorescence images. In the three schemes, the same architecture but the different training schemes were used. The Bonferroni-Holm method was used to correct for multiple testing of the three scheme comparisons. We also tested the performance of Scheme III and III’: in Scheme III’, ensemble learning and median filtering were added as the post-processing of Scheme III.

#### 2.5.1. Evaluation Metrics

We used the mean accuracy and $F_{1}$ value as the evaluation metrics to quantitatively assess the learning results. These metrics were given by

(4) $$mean\;accuracy=\frac{sensitivity+specificity}{2},$$

(5) $$F_{1}\;\mathrm{value}=\frac{2*sensitivity*precision}{sensitivity+specificity},$$

(6) $$precision=\frac{TP}{TP+FP}.$$

The values of sensitivity and precision were calculated using all images in one fold test set (see Section 2.5.2 and Section 2.5.3), because the values of TP and FN become 0 when nerves are not included in a test image. We compared the outputs from the models trained on the same data while using the paired t-test (two-sided test).

In all evaluation metrics, $F_{1}$ value is a prime metric for assessing the nerve segmentation performance. $F_{1}$ value, called Dice coefficient, is a harmonic mean of sensitivity (recall) and precision. The higher value of both metrics is required to extract nerves without excess or deficiency. Although all evaluation metrics used in this paper are shown in Results, $F_{1}$ value mainly indicates the performance of nerve segmentation.

#### 2.5.2. Evaluation of the Model Trained on Fluorescence Images

The model trained on the fluorescence images was evaluated by 10-fold cross validation. The 1818 fluorescence images were split into 10 folds randomly. We held back one fold as the test set, which was not used for training. Another fold and the remaining eight folds were used as the validation and training sets, respectively. The training was repeated until each of the nine folds were utilized as the validation set; nine models were prepared, where each model was trained on a slightly different data set. The segmentation performance was evaluated with the average of the above-mentioned metrics for the nine models.

#### 2.5.3. Evaluation of the Model Trained on the Coherent Anti-Stokes Raman Scattering Rigid Endoscopy Images (Scheme I, II, III)

The models that were trained on the CARS rigid endoscopy images were statistically evaluated by 8-fold nested cross validation [20]. The 24 CARS endoscopy images were split into eight folds randomly. In nested cross validation, the model was trained until each of the eight folds (including the test set) had served as the validation and test sets; 56 models were prepared. The segmentation performance was evaluated with the average and standard deviation of the above metrics for the 56 models.

#### 2.5.4. Ensemble Learning and Median Filter for Further Performance Improvement (Scheme III’)

**Figure 2:**
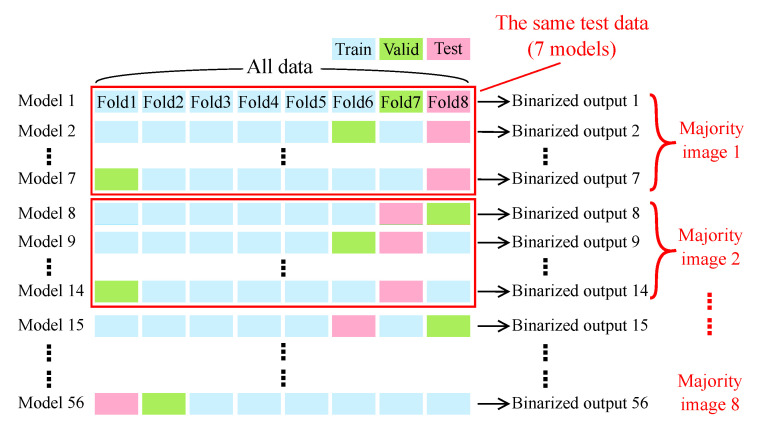
Overview of ensemble learning (majority voting strategy).

After the training of the model, ensemble learning (majority voting strategy) and a median filter were applied to further improve the performance. Figure 2 shows an overview of ensemble learning. After the eight-fold nested cross validation with CARS rigid endoscopy images, a total of 56 models with different weight sets (Models 1–56) were provided. The 56 models were divided into eight groups (a group is surrounded with a red box). The seven models in each group were trained with the same dataset, where the combination of training and validation sets was different. A final output of an ensemble model was decided by a majority decision of the seven binarized outputs pixel by pixel (Majority images 1–8) and filtered by a median filter with a 37 × 37 kernel.

## 3. Results

### 3.1. Fluorescence Images

**Figure 3:**
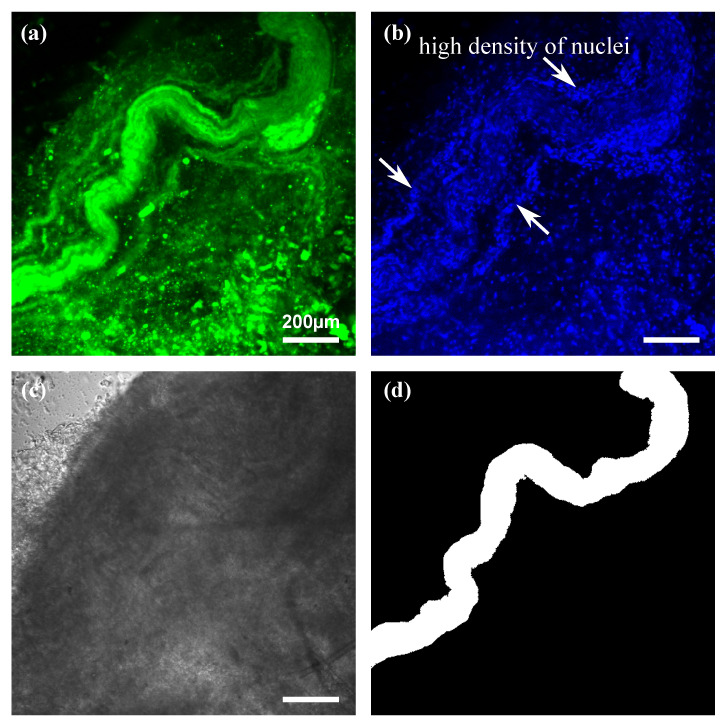
Confocal fluorescence microscopy imaging of rabbit peri-prostatic nerve. (**a**) FluoroMyelin${}^{\mathrm{TM}}$ Green image. (**b**) Hoechst image. (**c**) Transmission image. (**d**) Ground truth image prepared based on fluorescence images. Scale bar is 200 μm.

An imaging result with confocal fluorescence microscopy is shown in Figure 3. The four images show the same region in the sample, corresponding to a FluoroMyelin${}^{\mathrm{TM}}$ Green image (Figure 3a), a Hoechst image (Figure 3b), a transmission image (Figure 3c), and a ground truth image (Figure 3d) for pre-training on fluorescence images. The FluoroMyelin Green image clearly showed nerves; however, lipid-derived tissue from non-nerve tissues was also visualized. The ground truth images were manually prepared by removing non-nerve tissues from the FluoroMyelin Green images and binarizing the images. The blood vessels and nerves in the neurovascular bundle were distinguished by comparing the FluoroMyelin${}^{\mathrm{TM}}$ Green image with the Hoechst image based on the difference of the nuclei density indicated by white arrowheads. Lipid droplets and adipocytes were distinguished from nerves by shape.

### 3.2. Coherent Anti-Stokes Raman Scattering Rigid Endoscopy Images

**Figure 4:**
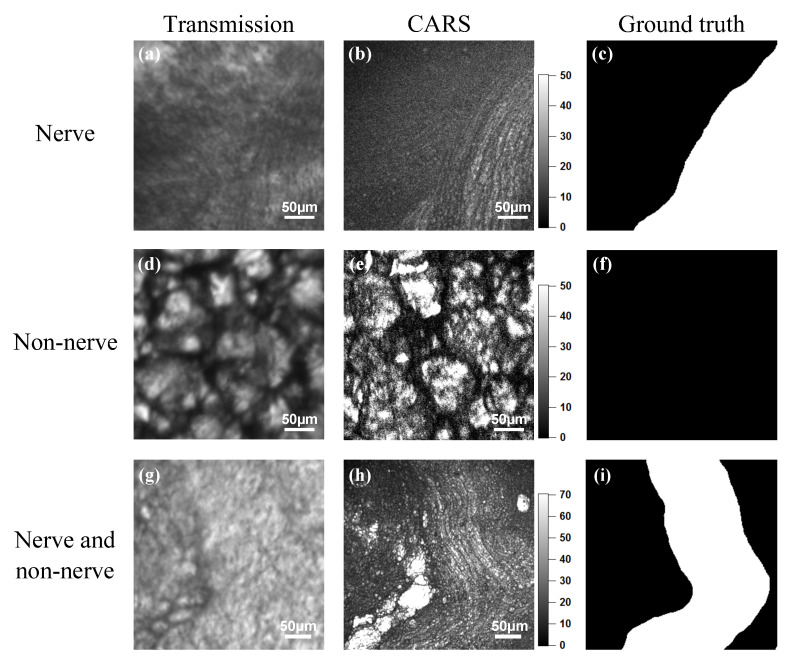
CARS endoscopic imaging of rabbit peri-prostatic fascia. (**a**,**d**,**g**) Transmission images. (**b**,**e**,**h**) CARS rigid endoscopy images. (**c**,**f**,**i**) Ground truth images. (**a**–**c**) A sample containing only nerves emitting large CARS signals. (**d**–**f**) A sample containing only non-nerve tissue emitting strong CARS signals. (**g**–**i**) A sample containing both nerves and non-nerve tissues emitting CARS signals.

Figure 4 shows the results of CARS endoscopic imaging of the non-labeled rabbit peri-prostatic fascia. The left, center, and right columns show the transmission images (Figure 4a,d,g), the CARS rigid endoscopy images (Figure 4b,e,h), and the ground truth images (Figure 4c,f,i), respectively. Bright fibrous structures are shown in Figure 4b, but not in Figure 4a. In Figure 4d,e, bright spherical structures appear. Figure 4h presents both bright fibrous and spherical structures. We considered that the bright fibrous structures indicated nerves, and the spherical structures indicated non-nerve tissues. In transmission images for CARS endoscopy (Figure 4a,g) as well as fluorescence microscopy (Figure 3c), the nerves are unclear since nerves are almost transparent. It is found that the contrast enhancement of nerves is needed to identify nerve positions. Figure 4c,f,i are ground truth images for nerve segmentation, where white pixels indicate nerves.

### 3.3. Nerve Segmentation with Deep Neural Network

**Figure 5:**
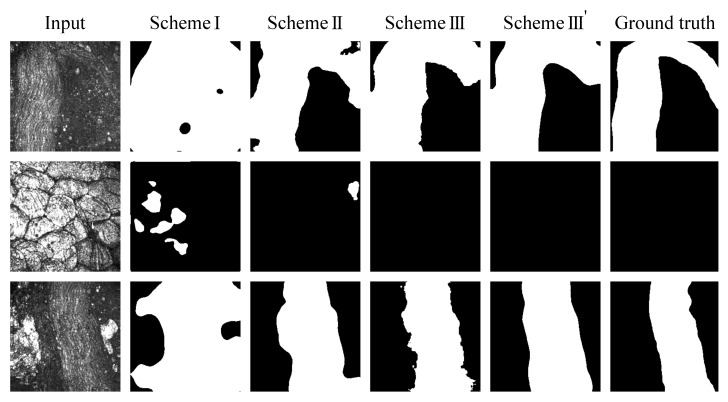
Results of nerve segmentation with four learning schemes.

Figure 5 shows the results of nerve segmentation with three learning schemes. The Input column shows the test images of the CARS rigid endoscopy. The Scheme I column shows the output images from the model trained on CARS rigid endoscopy images from scratch. The Scheme II column presents the images from the U-Net with the VGG16 encoder model whose decoder part was trained on CARS rigid endoscopy images. The Scheme III column displays the images from the U-Net with the VGG16 encoder model whose decoder part was pre-trained on fluorescence images and fine-tuned on CARS rigid endoscopy images. The Scheme III’ column shows the images that were obtained by applying ensemble learning and the median filtering to the images of Scheme III. The Ground truth column indicates the ground truth images. In all figures for Scheme I, many regions were predicted to be nerves. However, conversely, there were cases where many regions were predicted to be non-nerve tissues (not shown), and the predictions were biased towards either side. It was found that the nerves were correctly extracted by applying the VGG16 encoder (Scheme II) and further using the fluorescence images (Scheme III). The post-processing (Scheme III’) provided smooth region extraction.

Table 1 shows the results of statistical evaluation of the performance of the deep learning model. As evaluation metrics, the mean accuracy and $F_{1}$ value, and sensitivity, specificity, and precision are also shown. All of the evaluation metrics of Scheme II were significantly higher than those of Scheme I (Bonferroni-Holm corrected significance level: p=0.0167 for specificity and p=0.0250 for the other metrics). The metrics of Scheme III were also significantly higher than those of Scheme I (Bonferroni-Holm corrected significance level: p=0.0250 for specificity and p=0.0167 for the other metrics). The performance was improved by using VGG16 as the encoder part. The sensitivity, specificity and precision of Scheme III tended to be higher than those of Scheme II. For comparison of Scheme I, II and III, the mean accuracy and $F_{1}$ value of Scheme III were significantly higher than those of Scheme II (Bonferroni-Holm corrected significance level: p=0.05). The performance was further enhanced by using fluorescence images for pre-training in addition to the VGG16 encoder part. Although Scheme III’ were no-significant differences from Scheme III, all of the evaluation metrics and the standard deviations of Scheme III’ were better than those of Scheme III. In terms of $F_{1}$ value, pre-training on the fluorescence images (Scheme III) were significantly higher performance compared using VGG16 encoder part only (Scheme II).

## 4. Discussion

We demonstrated the nerve segmentation of CARS endoscopic images using U-Net as a deep learning model. Using the VGG16 encoder part pre-trained on ImageNet improved the performance of nerve segmentation. The improvement of the performance with transfer learning is similar to that in a previous report [15]. Pre-training on fluorescence images further improved the performance. Finally, a mean accuracy of 0.962 and an $F_{1}$ value of 0.860 were achieved with ensemble learning and median filtering. All nerve predictions showed a tendency to output the larger area comparing with the nerves region of the ground truth images. We consider that the tendency is preferable to predicting the narrower area for nerve preservation.

We examined the effect of pre-training with fluorescence images. Fluorescence images also visualize the lipid distribution, and they provide similar images to CARS. We considered the possibility of complementing a small dataset of CARS images with fluorescence images. Consequently, improved nerve segmentation performance was demonstrated by pre-training with fluorescence images. Such pre-training using fluorescence images might be useful for diagnostic applications as well as nerve segmentation. The specificity of label-free CARS images is not as high as that of immunostaining with fluorescent dyes. Therefore, deep learning models pre-trained with specially stained fluorescence images have the potential to further improve the specificity of label-free CARS images. Besides, fluorescence images will improve the truthness of ground truth images compared with present manual preparation from CARS images. Further researches are required using more accurate ground truth images obtained by staining and observing samples after CARS imaging.

Automated nerve segmentation from label-free CARS rigid endoscopic images with deep learning was achieved with remarkably high performance. Because CARS images visualize lipids, they visualize not only nerves, but also other tissues. The values of these evaluation metrics indicate that automated nerve segmentation correctly predicted whether a single CARS image contained nerves. We consider that it has sufficient cognitive ability for intraoperative nerve observation. We believe that the results of automated nerve segmentation from CARS imaging will contribute to intraoperative nerve identification without imposing additional strain on surgeons.

Using more models with ensemble learning will improve the performance of nerve segmentation. The results of the majority decision that are shown in Table 1 were examined with seven models to statistically evaluate the metrics. In the actual implementation, we can use 56 models for the majority decision, which will improve the performance. The required processing time was 549 ms for CARS nerve segmentation imaging with the majority decision of 56 models using the present PC environment. Therefore, it is speculated that the performance of the majority decision method is higher than the present results within a time sufficiently short not to interfere with surgery.

One of the other problems for intraoperative nerve identification is the long acquisition time of the CARS endoscopic images. The several minutes needed for CARS endoscopic imaging during surgery may not be very realistic, because of the burden on the patient and surgeon. We believe that nerve segmentation from CARS imaging with a low signal-to-noise ratio acquired with a shorter exposure time (several seconds) will realize intraoperative nerve imaging for safe nerve-sparing surgery.

## Figures and Tables

**Table 1:** Evaluation results of nerve segmentation with four learning schemes.

Evaluation Metric	Learning Scheme				*p* Value			
	I	II	III	III’	I vs. II	I vs. III	II vs. III	III vs. III’
*sensitivity*	0.689 ± 0.391	0.949 ± 0.056	0.962 ± 0.054	0.977 ± 0.025	<0.01	<0.01	0.14	0.16
*specificity*	0.843 ± 0.147	0.930 ± 0.046	0.937 ± 0.054	0.947 ± 0.030	<0.01	<0.01	0.33	0.19
*precision*	0.469 ± 0.239	0.719 ± 0.123	0.752 ± 0.118	0.772 ± 0.061	<0.01	<0.01	0.06	0.13
*mean accuracy*	0.766 ± 0.156	0.939 ± 0.026	0.950 ± 0.031	0.962 ± 0.014	<0.01	<0.01	0.03	0.06
$F_{1}$ value	0.469 ± 0.243	0.809 ± 0.083	0.837 ± 0.085	0.860 ± 0.034	<0.01	<0.01	0.03	0.05
